# Differences in HIV-related behaviors at Lugufu refugee camp and surrounding host villages, Tanzania

**DOI:** 10.1186/1752-1505-2-13

**Published:** 2008-10-17

**Authors:** Elizabeth A Rowley, Paul B Spiegel, Zawadi Tunze, Godfrey Mbaruku, Marian Schilperoord, Patterson Njogu

**Affiliations:** 1 Johns Hopkins Bloomberg School of Public Health, Baltimore, MD, USA; 2 UNHCR, Geneva, Switzerland; 3 Tanzania Red Cross National Society, Dar es Salaam, Tanzania; 4 Maweni Regional Hospital, Dar es Salaam, Tanzania

## Abstract

**Background:**

An HIV behavioral surveillance survey was undertaken in November 2005 at Lugufu refugee camp and surrounding host villages, located near western Tanzania's border with the Democratic Republic of Congo (DRC).

**Methods:**

The sample size was 1,743 persons based on cluster survey methodology. All members of selected households between 15–49 years old were eligible respondents. Questions included HIV-related behaviors, population displacement, mobility, networking and forced sex. Data was analyzed using Stata to measure differences in proportions (chi-square) and differences in means (t-test) between gender, age groups, and settlement location for variables of interest.

**Results:**

Study results reflect the complexity of factors that may promote or inhibit HIV transmission in conflict-affected and displaced populations. Within this setting, factors that may increase the risk of HIV infections among refugees compared to the population in surrounding villages include young age of sexual initiation among males (15.9 years vs. 19.8 years, p = .000), high-risk sex partners in the 15–24 year age group (40% vs. 21%, χ^2 ^33.83, p = .000), limited access to income (16% vs. 51% χ^2 ^222.94, p = .000), and the vulnerability of refugee women, especially widowed, divorced and never-married women, to transactional sex (married vs. never married, divorced, widowed: for 15–24 age group, 4% and 18% respectively, χ^2 ^8.07, p = .004; for 25–49 age group, 4% and 23% respectively, χ^2 ^21.46, p = .000). A majority of both refugee and host village respondents who experienced forced sex in the past 12 months identified their partner as perpetrator (64% camp and 87% in villages). Although restrictions on movements in and out of the camp exist, there was regular interaction between communities. Condom use was found to be below 50%, and expanded population networks may also increase opportunities for HIV transmission. Availability of refugee health services may be a protective factor. Most respondents knew where to go for HIV testing (84% of refugee respondents and 78% of respondents in surrounding villages), while more refugees than respondents from villages had ever been tested (42% vs. 22%, χ^2 ^63.69, p = .000).

**Conclusion:**

This research has important programmatic implications. Regardless of differences between camp and village populations, study results point to the need for targeted activities within each population. Services should include youth education and life skills programs emphasizing the benefits of delayed sexual initiation and the risks involved in transactional sex, especially in the camp where greater proportions of youth are affected by these issues relative to the surrounding host villages. As well, programs should stress the importance of correct and consistent condom use to increase usage in both populations. Further investigation into forced sex within regular partnerships, and programs that encourage male involvement in addressing this issue are needed. Program managers should verify that current commodity distribution systems ensure vulnerable women's access to resources, and consider additional program responses.

## Background

Available data indicate that while 10% of the world's population lives in Africa, the continent is home to 68% of adults and 90% of children living with HIV/AIDS [1,2]. Over the last sixty years, Africa has also witnessed a greater number of conflicts than any other region in the world [3]. Conflict and displacement can lead to elevated infectious disease and nutrition-related mortality and morbidity, though the public health field has made progress against the health impact of complex emergencies [4]. While population mobility and poverty could exacerbate the spread of HIV in conflict-affected populations, research in recent years demonstrates the complexities of infection dynamics in such settings [5-7]. Factors that can increase HIV transmission include the breakdown of social structures, limited access to income, vulnerability to rape and transactional sex, and reduced health resources. However, conflict can also promote protective factors such as reduced population mobility, increased geographic isolation, and the possibility of better access to services after displacement compared to area of origin. The length of time that a population has been displaced and the HIV prevalence levels in both area of origin and area of displacement are major determinants of whether the above factors lead to an increase or decrease in HIV infection rates [6].

In 1999, the Great Lakes Initiative on AIDS (GLIA) was launched as a regional initiative in Burundi, the Democratic Republic of Congo (DRC), Kenya, Rwanda, Tanzania, and Uganda. Today, GLIA functions through the National AIDS Commissions of these countries to reduce HIV infections and mitigate the socio-economic impact of the epidemic. As part of this initiative, the United Nations High Commissioner for Refugees (UNHCR), GLIA, and other partners have undertaken HIV behavioral surveillance surveys (BSS) in most of the six countries. The surveys collect baseline data for HIV prevention program activities. The survey design is based on the Family Health International BSS model, with the addition of specific questions on population displacement, mobility and networking, and sexual and gender based violence (SGBV). A unique feature of the survey is its application in both refugee camps and surrounding villages, allowing for a better understanding of differences, similarities and interactions between populations. The survey was carried out in November 2005 through the National AIDS Control Program of Tanzania (NACP), the Tanzania Commission for AIDS and UNHCR. The Tanzania National Medical Institute provided approval for the study. The objectives of this article are to report on important factors that affect HIV transmission, examine accessibility and utilization of specific HIV interventions, and provide recommendations to improve HIV programs among refugees in Lugufu camp and the surrounding host populations in Tanzania.

Tanzania's first cases of AIDS were reported in 1983 from Kagera Region and current estimates are that 6.3% of men and 7.7% of women in the country are HIV-positive [8]. Up to 180,000 Tanzanians have died from the virus [9]. As of June 2005, Tanzania was host to over 400,000 Burundian and 150,000 Congolese refugees, and a smaller number of Rwandans [10]. The Lugufu refugee camp population was estimated to be 22,968 households (94,417 persons) at the time of fieldwork and was almost exclusively Congolese. The camp was established in 1997 and is located in a remote area of Kigoma District in western Tanzania. The Tanzanian Red Cross Society, supported by UNHCR, provides health services in the camp including HIV testing and counseling. Uvinza and Kazulamimba villages were selected as local population settlement survey sites. Uvinza is composed of 12 sub-villages with a total population of 2,109 households. Kazulamimba includes 11 sub-villages and a total population of 2,660 households. Each village lies about 25 km. from the Lugufu camp area. Local security regulations limit the movement of populations between the camp and surrounding villages. The most recent HIV prevalence estimates for camp populations at Lugufu and Nyaragusu (1% in 2001, 2.5% in 2002, and 1.8% in 2003 based on antenatal care sentinel surveillance data) are lower than for Kigoma region which in 2003 had a population-based HIV prevalence measure of 7% [7,8].

## Methods

Sample size took into account cluster sampling with a design effect of 2, and was based on prevalence measures for two key HIV-related behavioral indicators: proportion of respondents aged 15–24 years reporting condom use at last sexual intercourse with a non-regular sexual partner; and proportion of respondents aged 15–24 years who could correctly identify ways of preventing the sexual transmission of HIV and who reject major misconceptions about HIV transmission or prevention. A sample size of 1600 respondents (800 in Lugufu camp and 800 in the surrounding villages) was determined using the two-sample comparison of proportions formula to measure change of at least 15% between baseline and final surveys, with a precision level of .05, power of .20, and 50% initial prevalence of the selected indicators.

Systematic sampling was used in Lugufu camp based on UNHCR household listings. Two factors necessitated re-sampling. First, repatriation exercises were ongoing during the survey and several households on the listing had recently repatriated. Second, in some cases refugees had shifted residence from one part of the camp to another between registration and verification exercises and could not be located. Re-sampling was undertaken as necessary after removing absent households from the listing.

In Uvinza and Kazulamimba, data on the total number of households per sub-village, but not complete household listings, were available. All sub-villages within each village were included. Cluster sampling was employed with a target number of households per sub-village determined proportional to population size. Household selection was made using a random start method.

All members of selected households between 15–49 years old were eligible respondents. A household member was defined as anyone living and sharing meals with the household for at least two weeks. In the case of polygamous men maintaining more than one household, only households previously identified through the sampling methods described above were included. If more than one family lived in the same household compound, they were interviewed as two separate households.

Interviewers revisited households and/or individuals within households at least three times before coding as absent. Households confirmed to be unoccupied for four weeks or more were marked as either abandoned or on extended travel depending on circumstances. For sampling purposes, interviewers did not replace households. Suitable interviewers were selected by UNHCR for camp-based interviews (36 enumerators) and by local NACP representatives for village interviews (29 enumerators). Interviewers received a three-day training including practice exercises. Interviews in the camp and villages were conducted in Kiswahili which is spoken by both Congolese refugees and Tanzanians. Interviewers visited households in male-female pairs so that respondents could be interviewed by someone of their own gender if desired. Verbal consent was obtained prior to all interviews and clearly documented. For respondents under 18 years, consent first was obtained from the head of household and/or other household member aged 18 years or above. Absences and refusals were recorded.

## Results

A total of 802 interviews were completed in Lugufu camp and 941 in the two surrounding villages. At the household level, non-participation due to absence (households abandoned, repatriated, or on extended travel), ineligibility (households without eligible members), and refusal was 0.8% in Lugufu and 2% in villages. Total non-participation of household members due to absence, refusal or other reasons, within households where other members were interviewed, was 11% in the camp and 9% in the villages; this was primarily due to household member absence. In the camp, a larger proportion of household members who could not be interviewed were female (55%) compared to males (45%). In surrounding villages this was 44% and 56%, respectively. The percentage of household members who refused, within households where other members were interviewed, was 0 in the camp and 0.1% in the villages.

### Background characteristics

Camp respondents, especially males, were younger than those in surrounding villages with 48% of males in the camps being in the 15–24 year age group compared to 30% of male village respondents (χ^2 ^24.5, p = .000). Most respondents in both settings were married at time of interview followed by never married; a larger proportion of female camp respondents were widowed (8%) compared to the local population (2%) (χ^2 ^16.95, p = .000). Half of all camp respondents were of protestant denominations, while 48% in villages were Muslim. Refugees compared with villagers, and males in both settings compared with females, had higher education (secondary school and above for both genders: 52% in camp vs. 7% in villages, χ^2 ^409.28, p = .000; in the camp: secondary school and above 81% for males vs. 27% for females, χ^2 ^221.96, p = .000; in the villages: 11% for males vs. 5% for females, χ^2 ^9.49, p = .002). Many more respondents from villages (51%) had access to income generation opportunities, mainly in agriculture, compared with camp respondents (16%) (χ^2 ^222.94, p = .000) (Table 1).

**Table 1:** Background characteristics of respondents

**Characteristic**	**Refugee camp (n = 761)**			**Surrounding host villages (n = 929)**			
	**Male **%	**Female** %	**Total** %	**Male** %	**Female** %	**Total** %	**χ^2 ^p- value^a^**
**Age (years)**							
Total	n = 352	n = 409	n = 761	n = 381	n = 548	n = 929	n = 1690

15–19	30	24	27	19	18	18	
20–24	18	18	18	11	25	19	16.87
25–49	52	58	55	70	57	62	p = .000

**Marital status**							
Total	n = 349	n = 407	n = 756	n = 377	n = 545	n = 922	n = 1678

Currently married	53	57	55	65	64	65	
Never married	45	23	33	30	23.5	26	23.74
Divorced	2	12	7	5	10	8	p = .000
Widow/widower	.25	8	4	1	2	2	

**Religion**							
Total	n = 348	n = 404	n = 752	n = 381	n = 545	n = 926	n = 1679

Catholic	27	27	27	29	23	25	
Protestant	48	51	50	23	22	23	298.02
Muslim	13	10	11	45	51	48	p = .000
Other	12	11	11	3	5	4	

**Education**							
Total	n = 351	n = 408	n = 759	n = 381	n = 546	n = 927	n = 1685

Never attended school	3	25	15	9	19	15	
Did not complete full grade/level	2	6	4	7	11	10	442.79
Primary completed	14	42	29	73	64	68	p = .000
Secondary school and above	81	27	52	11	5	7	

**Employment**							
Total	n = 350	n = 408	n = 758	n = 378	n = 544	n = 922	n = 1680

Employed^a^	21	12.5	16	56	48	51	222.94
Unemployed	79	87.5	84	44	52	49	p = .000

^a ^Refers to categories of employment including agriculture, trade, pastoralism, transport, fishing, crafts, private services, public services, humanitarian and development, and other.

^b ^Reported χ^2 ^and p-value for difference in total proportions refugees vs. nationals.

Data on several core indicators describe key sexual behaviors, health service utilization, and knowledge about HIV/AIDS. These indicators follow internationally accepted HIV indicators and focus on persons aged 15–24 years. Core indicators specific for these populations, including information about forced sex, displacement and mobility were also included (Table 2).

**Table 2:** Core indicators

**Characteristic**	**Refugee camp**			**Surrounding host villages**			
	**Male %**	**Female %**	**Total %**	**Male %**	**Female %**	**Total %**	**χ^2 ^p-value^a^**
**Sexual behavior**							

Never-married young people aged 15–24 who have never had sex	21	52	32	65	48	56	24.23
	n = 141	n = 81	n = 222	n = 92	n = 111	n = 203	p = .000

Never-married young people aged 15–24 who have abstained from sexual intercourse for the past 12 months	29	56	39	70	57	63	21.05
	n = 123	n = 75	n = 198	n = 86	n = 95	n = 181	p = .000

Sex with a non-regular partner in the last 12 months among men and women aged 15–24^b^	50	28	39	18	21	20	31.54
	n = 169	n = 172	n = 341	n = 115	n = 234	n = 349	p = .000

Condom use at last sex with a non-regular partner among men and women aged 15–24	36	44	39	38	24	28	2.24
	n = 85	n = 48	n = 133	n = 21	n = 46	n = 67	p = .134

Sex with a transactional partner in the last 12 months among men and women aged 15–24^c^	21	12	16	8	1	3	32.02
	n = 167	n = 169	n = 336	n = 114	n = 232	n = 346	p = .000

Condom use at last sex with a transactional partner among men and women aged 15–24	49	35	44	22	33	25	1.42
	n = 35	n = 20	n = 55	n = 9	n = 3	n = 12	p = .233

High risk sex in past 12 months among men and women aged 15–24^d^	53	28	40	22	20	21	33.83
	n = 169	n = 172	n = 341	n = 115	n = 234	n = 349	p = .000

Condom use at last high risk sex among men and women aged 15–24	36	44	39	32	24	27	2.94
	n = 89	n = 48	n = 137	n = 25	n = 46	n = 71	p = .086

More than one sex partner in past 12 months among men and women aged 15–49	50	26	37	36	27	30	8.17
	n = 352	n = 409	n = 761	n = 381	n = 548	n = 929	p = .004

**HIV testing**							

Had an HIV test in the past 12 months and received the results, among men and women aged 15–49	19	17	18	10	11	10	.37
	n = 352	n = 409	n = 761	n = 381	n = 548	n = 929	p = .544

**STI health facility utilization**							

Had an STI symptom in the past 12 months and sought treatment at a health facility, among men and women aged 15–49	50	63	57	88	86	87	7.57
	n = 22	n = 32	n = 54	n = 16	n = 14	n = 30	p = .006

**Knowledge, attitudes and misconceptions**							

Comprehensive correct knowledge of HIV/AIDS among men and women aged 15–24^e^	25	26	26	32	35	34	6.07
	n = 169	n = 172	n = 341	n = 115	n = 234	n = 349	p = .014

Accepting attitudes towards people living with HIV/AIDS among men and women aged 15–49^f^	10	8	9	24	24	24	62.22
	n = 322	n = 382	n = 704	n = 360	n = 532	n = 892	p = .000

**Displacement situations**							

Percent of women aged 15–49 who were forced to have sex in the past 12 months	---	3	---	---	1	---	2.53
		n = 409			n = 548		p = .112

Men and women aged 15–49 residing in current community for less than 12 months	.28	1	1	6	7	7	38.37
	n = 352	n = 409	n = 761	n = 381	n = 548	n = 929	p = .000

Away from home for four or more consecutive weeks in past 12 months among men and women aged 15–49	34	13	22	19	14	17	9.27
	n = 352	n = 408	n = 760	n = 380	n = 547	n = 927	p = .002

Men and women aged 15–49 years who visit surrounding community at least once a month	24	10	17	28	19	23	9.61
	n = 352	n = 409	n = 761	n = 381	n = 548	n = 929	p = .002

^a ^Reported χ^2 ^and p-value for difference in total proportions refugees vs. nationals.

^b ^A non-regular partner is defined as any sexual partner different from the one the respondent lives with or is married to and from whom the respondent does not receive or give money, gifts, or favors.

^c ^A transactional partner is defined as a sexual partner with whom the respondent exchanged sex for money, gifts, or favors.

^d ^High risk sex is defined as sex with a non-regular or transactional sex partner.

^e ^Respondents have comprehensive and correct knowledge of HIV if they correctly identified two major ways of preventing HIV sexual transmission (using condoms and limiting sex to one faithful, uninfected partner), and if they rejected two common misconceptions (mosquitoes transmit HIV, sharing food with an infected person transmits HIV), and if they knew that a healthy-looking person can transmit HIV.

^f ^Respondents have accepting attitudes if they reported to be willing to care for a family member sick with AIDS in their own household, and would buy vegetables from a shopkeeper with AIDS, and feel a teacher with HIV should be allowed to continue working, and do not feel that it should be kept a secret if a family member has HIV.

Nearly all respondents in both the camp and villages had previously heard about HIV/AIDS (97% and 98%, respectively). These proportions did not differ greatly for the 15–24 year old group (96% and 98%, respectively). Most respondents in the camp (95%) and villages (91%) had heard about sexually transmitted infections (STIs). Of those who ever had a genital discharge, ulcer or sore, a greater percentage of camp (75%) than village respondents (67%), sought treatment, though the difference was not statistically significant.

### Sexual behaviors

Sexual behavior indicators varied greatly between the two populations and between genders within each population. A significantly greater proportion of never-married 15–24 year old respondents in the villages, compared with the camp, reported that they had never had sex (56% vs. 32%, χ^2 ^24.23, p = .0001). This difference was especially marked among males in villages compared with the camp (65% vs. 21%, χ^2 ^47.02, p = .000). Average age at first sexual intercourse for males in the camp was much lower than in villages (15.9 years vs. 19.8 years, t-test p = .000).

Among those never-married respondents aged 15–24 years who had ever had sex, a greater proportion in villages compared with the camp reported abstinence during the past 12 months (63% vs. 39%, χ^2 ^21.05, p = .000). Again, the largest difference was for males, who reported a significantly higher abstinence rate in the villages than in the camp (70% vs. 29%, χ^2 ^33.43, p = .000). The difference in abstinence between genders within location was significant only in the camp where 56% of unmarried females and 29% of unmarried males reported abstinence in the past 12 months (χ^2 ^13.94, p = .000).

High-risk sex, defined as sex with a non-regular partner (sexual partner different from the one the respondent lives with or is married to) or transactional sex partner (sexual partner with whom the respondent exchanged sex for money, gifts, or favors), during the last 12 months, was reported by a greater proportion of respondents 15–24 years in the camp compared with villages (40% vs. 21%, χ^2 ^33.83, p = .000); more males than females in the camp reported this behavior (53% vs. 28%, χ^2 ^25.72, p = .000). In this age group, transactional sex in the past 12 months was reported more frequently in the camp than villages (16% vs. 3%, χ^2 ^32.02, p = .000) and more frequently by males than females within both the camp (21% vs. 12%, χ^2 ^5.11, p = .024) and the villages (7% vs. 1%, χ^2 ^9.95, p = .002). In general, condom use at last sex by 15–24 year age group respondents was higher in the camp than villages, whether for a non-regular partner (39% vs. 20%), transactional sex partner (44% vs. 25%), or high-risk partner (40% vs. 21%). However, the total number of respondents in this age group who indicated sexual partners of these categories was small and differences between locations in condom use were not statistically significant.

### Displacement, mobility, and networking

Most refugees and villagers had lived in their community for over five years (79% and 78%, respectively). More local respondents than refugees reported living in the area for 12 months or less (0.8% in camp, 7% in villages, χ^2 ^38.37, p = .000), with minimal difference between genders within each population (Table 3). There was no meaningful difference between age groups.

**Table 3:** Indicators of displacement, mobility, and networking, all ages and 15–24 years

**Characteristic**	**Refugee camp**			**Surrounding host villages**			
	**Male %**	**Female %**	**Total %**	**Male %**	**Female %**	**Total %**	**χ^2 ^p-value^a^**
**Length of time living in current community**							

*All ages*	n = 348	n = 402	n = 750	n = 375	n = 539	n = 914	

Always	0	0	0	49	46	47	
< 6 months	0.3	1	.7	2	3	3	
6–12 months	0	.2	.1	4	4	4	607
1–2 years	1.4	2	2	5	5	5	p = .000
3–5 years	17	19	18	9	11	10	
> 5 years	82	77	79	31	31	31	

*15–24 years*	n = 167	n = 169	n = 336	n = 114	n = 230	n = 344	

Always	0	0	0	53	44	47	
< 6 months	0	2	1	3	4	3	
6–12 months	0	0	0	5	8	7	299.04
1–2 years	2	4	3	9	8	8	p = .000
3–5 years	20	22	21	6	13	11	
> 5 years	77	72	74	24	23	23	

**Away from home for 4 or more consecutive weeks within the last 12 months**							

*All ages*	34	13	22	19	14	17	9.27
	n = 352	n = 408	n = 760	n = 380	n = 548	n = 927	p = .002

*15–24 years*	33	12	22	17	16	16	3.51
	n = 169	n = 171	n = 340	n = 114	n = 233	n = 347	p = .061

**Frequency of visits to camp/surrounding community**							

*All ages*	n = 348	n = 404	n = 752	n = 381	n = 544	n = 925	

Never	58	81	70	62	70	67	
Less than once per month	18	9	13	10.5	10	10	17.30
Once a month	14	6	9	19	15	16.5	p = .001
Many times in a month	10	5	7	9	4	6	

*15–24 years*	n = 166	n = 169	n = 335	n = 115	n = 232	n = 347	

Never	60	82	71	71	74	73	
Less than once per month	20	8	14	8	8	8	8.77
Once a month	14	5	10	16	13	14	p = .032
Many times in a month	6	6	6	5	4	5	

^a ^Reported χ^2 ^and p-value for difference in total proportions refugees vs. nationals

In both the camp and villages, more males than females reported they had ever left their current residence for four weeks or more. Results for camp respondents were significant for all ages (34% males vs. 13% females, χ^2 ^49.40, p = .000), and the 15–24 year age group (33% males vs. 12% females, χ^2 ^21.49, p = .000). For village respondents, the difference between genders was smaller for all ages and of borderline significance (19% males vs. 14% females, χ^2 ^4.12, p = .042), and was insignificant for the 15–24 year age group (17% males vs. 16% females, χ^2 ^0.01, p = .933). Among those who had been away from home for at least one month in the previous 12 months, in both the camp and villages, the purpose of travel for most was family-related (63% and 54%, respectively).

The majority of respondents (70% in camp, 67% in villages) reported that they never go to the other community. Among camp respondents who do visit the villages, the largest proportion indicated this was less than once a month (13%), while more village respondents reported visiting the camp once a month (16.5%). Similar to the difference between genders noted above, more males than females in the camp indicated that they visited the surrounding villages once per month (14% males, 6% females) or many times per month (10% males, 5% females). Similar differences across gender are noted in the 15–24 year age group responses. Among refugees of all ages who reported visiting the villages, the dominant reasons for the last visit were shopping/market-related (62.5%) and trade (15.5%), without major differences by gender. For village respondents, the most frequently cited reasons were to visit a friend or relative (56%) and for shopping/market (27.5%).

### Transactional sex and forced sex

Questions related to transactional sex were asked of all respondents and did not distinguish whether the respondent paid for, or was paid for, the sexual transaction. A greater proportion of refugees compared with villagers indicated that they had ever had transactional sex (20% vs. 6%, χ^2 ^72.47, p = .000). A similar trend was noted among those who reported transactional sex within the past 12 months (14% vs. 4%, χ^2 ^51.14, p = .000). Among both refugee and village respondents, the difference between proportions in the 15–24 vs. 25–49 age groups was not significant (refugees: 23% vs. 18%, χ^2 ^2.63, p = .105; villagers: 7% vs. 6%, χ^2 ^.81, p = .369). The same was true for transactional sex within the past 12 months (refugees: 16% vs. 13%, χ^2 ^2.19, p = .138; villagers: 3% vs. 5%, χ^2 ^.98, p = .322).

Among female respondents in the camp, for both 15–24 and 25–49 year age groups, significantly greater proportions of those who reported transactional sex in the past 12 months were either never married, divorced, or widowed compared with those who were married (married vs. never married, divorced, widowed: for 15–24 age group 4% and 18% respectively, χ^2 ^8.07, p = .004; for 25–49 age group 4% and 23% respectively, χ^2 ^21.46, p = .000). In both the camp and villages, most respondents (both males and females, both age groups) reported limited access to income. There was no significant difference in reporting of income between those who indicated transactional sex in the past 12 months compared with those who did not. In the camp, the most common form of payment was money (64.5%), which in a few cases was combined with a gift (6.5%). In the villages, most respondents indicated exchange of sex for both money and a gift (57.5%).

Refugee respondents who ever had transactional sex were asked whether it occurred before, during or after displacement. These response categories were not exclusive; each respondent could answer to all that applied. The majority of responses (92%) indicated that transactional sex more often occurred after displacement than before or during. For village respondents as well, there were many more responses (90%) reporting transactional sex to have occurred after the arrival of refugees to the community than before.

Transactional sex was most frequently reported to have occurred within communities. Among those who ever had transactional sex, 82% of camp respondents reported that their transactional sex partner was a refugee, with a much smaller proportion (10%) indicating someone from the surrounding villages. Similarly, the majority of village respondents who ever had transactional sex indicated that the partner was from their own community (66%) rather than a refugee (11%). The difference between populations (camp vs. villages) in the proportion of respondents who indicated their last transactional sex partner was a refugee compared to someone from the local community was statistically significant (χ^2^82.91, p = .000) (Table 4).

**Table 4:** Transactional sex partner among those respondents who ever had transactional sex

**Characteristic**	**Refugee camp**			**Surrounding host villages**			
	**Male** %	**Female** %	**Total** %	**Male** %	**Female** %	**Total %**	**χ^2 ^p-value^a^**
Transactional sex partner							
Total	n = 79	n = 58	n = 137	n = 31	n = 22	n = 52	

Refugee	82	81	82	13	9	11	82.91
Person from local community	13	7	10	71	59	66	p = .000
							
Military, paramilitary, police	0	2	1	0	14	6	
Humanitarian/development worker	1	7	4	10	18	13	
Other	4	2	3	3	0	2	

Timing of transactional sex							---
Total^b^	n = 26	n = 39	n = 65	n = 14	n = 25	n = 39	

*Camp respondents*							

Before displacement	7	13	20	---	---	---	---
							
During displacement	5	7	12				
							
After displacement	14	19	33				

*Surrounding host village respondents*							

Before arrival of refugees	---	---	---	3	8	11	---
							
After arrival of refugees				11	17	28	

^a ^Reported χ^2 ^and p-value for difference in total proportions refugees vs. nationals for comparison of "refugee" and "person from local community" response categories.

^b ^Response categories not mutually exclusive; total refers to number of responses not respondents

Forced sex in this survey was defined through the question "have you ever been forced to have sex against your will?" The proportion of respondents who indicated ever experiencing forced sex was 10% in the camp and 4% in the villages (χ^2 ^16.44, p = .000) with similar proportions of males and females reporting forced sex (Table 5). Information about the timing of forced sex incidents among both camp and surrounding host village respondents indicates that most cases occurred after displacement. Less than 50% of those who ever experienced forced sex reported that it had happened within the past 12 months. The majority of respondents who experienced forced sex within the past 12 months reported that the perpetrator was their regular partner (64% camp and 87% in villages).

**Table 5:** Forced sex

**Characteristic**	**Refugee camp**			**Surrounding host villages**			
	**Male %**	**Female %**	**Total %**	**Male %**	**Female %**	**Total %**	**χ^2 ^p-value^a^**
**Experience of forced sex**							

Have been forced to have sex against will (Among those who ever had sex)	9	10	10	4	4	4	16.44
	n = 267	n = 298	n = 565	n = 334	n = 450	n = 784	p = .000

Have you been forced to have sex against your will in the past 12 months? (Among those who ever experienced forced sex)	50	41	45	50	38	43	.03
	n = 24	n = 32	n = 56	n = 14	n = 21	n = 35	p = .867

**Timing of forced sex**							
Total^b^	n = 26	n = 39	n = 65	n = 14	n = 25	n = 39	---

*Camp respondents*							

Before displacement	27	33	31	---	---	---	---
							
During displacement	18	18	18				
							
After displacement	54	49	51				

*Surrounding host village respondents*							

Before arrival of refugees	---	---	---	21	32	28	---
							
After arrival of refugees				79	68	72	

**Perpetrator of forced sex ** (Among those who experienced forced sex in the past 12 months)							
Total^b^	n = 12	n = 13	n = 25	n = 7	n = 8	n = 15	n = 40

Regular partner	42	85	64	85	88	87	2.42 p = .120

Other family member	17	8	12	0	12.5	7	.29 p = .586

Non-family member	42	15	28	14	0	7	2.67 p = .102

^a ^Reported χ^2 ^and p-value for difference in total proportions refugees vs. nationals

^b ^Response categories not mutually exclusive; total refers to number of responses not respondents

### Exposure and access to condoms and other HIV-prevention interventions

**Figure 1:**
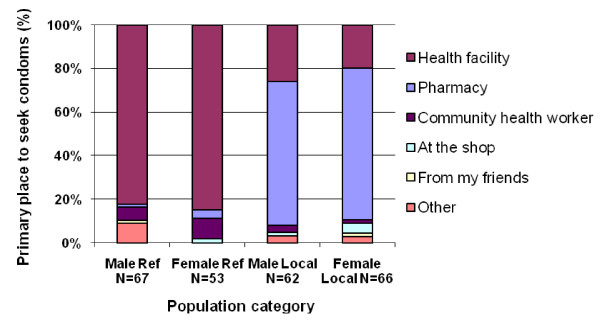
Primary place where respondents sought condoms: Refugee camp and surrounding host villages.

Almost all respondents in both the camp and villages indicated that they knew where to obtain condoms (95% and 97%, respectively). The majority of both male and female respondents (82% and 85%, respectively) in the camp reported that they first sought condoms from health facilities. In the villages, most respondents' first source was a pharmacy (66% for males, 70% for females) with a smaller proportion indicating that a health facility was the first place they went for condoms (Figure 1). Few camp respondents described other locations, though the community health worker was a source for both males (6%) and females (9%).

In both the camp and villages, 86% of respondents reported that they could get a condom every time it was needed. The reported accessibility of condoms did not vary significantly across age groups. Among those who reported constraints in obtaining condoms, the most common reason among female camp respondents was health workers' attitudes, while among male camp respondents it was facilities not being open at convenient hours and fear of being seen obtaining condoms. In the villages, for males, the main reason was fear of being seen, while some respondents highlighted cost. For women, the distance to, and working hours of places where one can get a condom, as well as cost, were reasons provided.

Among both camp and village respondents there was high awareness of where to go for HIV testing, though this was higher in the camp (84%) than in villages (78%) (χ^2 ^23.47, p = .000). More camp respondents (42%) than respondents from villages (22%) had ever tested for HIV (χ^2 ^63.69, p = .000). Among village and camp respondents, the proportions that tested within the past 12 months were similar, without major differences across age groups. A greater percentage of camp respondents who had an HIV test within the past 12 months received pre-test counseling (95% for all ages combined) compared with village respondents (76%) (χ^2 ^19.46, p = .000).

## Discussion

Survey results show large differences between the camp and surrounding village populations in several important indicators. Programmatic recommendations must be based on the needs, risks and characteristics of individual populations, as well as comparisons between populations, due to the relatively significant level of interaction among them. The data indicate that within younger age groups, refugees had an earlier sexual debut than local populations as marked by average age at sexual debut, especially for males. Though not a direct comparison, it is interesting to note that the national median age at first sex for males in the 20–24 year age group at this time was 18.3 years [8], higher than the average age for refugee males aged 15–24 (15.9 years), but lower than in the villages included in this study (19.8 years), Several factors could account for the lower age at sexual debut among refugees compared to village respondents, including underlying differences between the two populations in sexual initiation customs, marriage and childbearing preferences, changes in social norms that may accompany prolonged displacement, or differences in stigma related to premarital sex and respondent willingness to discuss this issue in a survey. Qualitative research is necessary to better understand the dynamics of sexual debut in this context.

That a greater proportion of respondents reported high-risk sex in the camp than in the villages may point to behavioral changes as a result of displacement and increased vulnerabilities within this population; however, there are no baseline data for comparison. Given village respondents' greater access to income relative to camp respondents, it is perhaps surprising that very little transactional sex was reported between refugee and village communities. Explanations could include sufficient supply and demand for transactional sex within populations to preclude the need to go outside one's community, or the populations may be uncomfortable in undertaking transactional sex arrangements with persons from an "unknown" community. Further qualitative investigation into this question is necessary, as it is often assumed that host populations with greater means may exploit refugee populations of lesser means. Although the proportion of younger respondents who ever had transactional sex was not significantly higher than older respondents for either location, it is of concern that 16% of young refugees reported transactional sex within the past 12 months, including more than one in every five young males. This proportion is high compared to both youth in the surrounding villages who reported transactional sex (3%) and the national average for 15–24 year old males (12.7%) who either paid for sex in the past 12 months or reported a commercial sex worker as at least one of their last three sexual partners in the past 12 months [8]. Younger refugees, particularly young males, need targeted programs to reduce this high risk behavior. Refugee women of all ages who were never married, divorced or widowed experienced transactional sex in significantly greater proportions than currently married women, and interventions targeted to reduce this vulnerability are required.

A somewhat unanticipated result of this research was that although most respondents in both the camp and surrounding villages had lived in their communities more than five years, a greater proportion of village respondents compared with refugees had been living there for less than 12 months. This points to the relative stability of this refugee population and the possibility that newcomers to host villages were attracted by services and work opportunities offered by international organizations in the area. Although the majority of both refugee and village respondents do not leave their communities for extended periods, the proportions that do, 22% and 17% respectively, are not small and indicate substantial interaction with outside environments. Results also show that while official restrictions on movements in and out of the camp exist, there is regular interaction between refugees and villagers for at least one third of both populations, primarily for economic activities. Isolation of communities and restrictions on interaction between communities, that could be a protective factor against HIV transmission, seem to be less applicable in this context. The greater mobility of refugee males compared with females, and their greater interaction with villagers, could widen sexual networks and influence sexual behaviors differently from what is observed within the camp. Further analysis of existing data and more focused research could generate a better understanding about HIV-related behaviors among mobile versus less mobile refugee males and females.

Forced sex in conflict-affected populations is typically assumed to be perpetrated against women by men in national military forces, armed insurgencies or criminal groups, as is occurring now in the DRC [11-13] and Chad [14,15]. The results of this survey show that the majority of both refugee and village respondents who had experienced forced sex within the previous 12 months identified their regular partner as perpetrator. Although the majority of refugees who experienced forced sex indicated this occurred either during or after displacement, a relatively small number of refugees reported forced sex by someone outside the family. While this may be due to misinterpretation of the question, or respondents' unwillingness to report such incidents, it may also reflect a stable post emergency situation where domestic violence is a major factor. This highlights the complexity of SGBV in general, and in conflict settings where intimate partner violence might increase during and after forced migration due to changes in gender power structures and a sense of powerlessness among males. In recent years, more attention has been focused on this issue [16,17], but it remains poorly understood. There is limited research on intimate partner violence in refugee settings and often insufficient programmatic attention.

It is encouraging that comparatively high proportions of individuals engaged in high-risk sex and transactional sex, particularly in the 15–24 year age group, reported condom use at the last such sexual encounter within the past 12 months. Comparison with 2004 Tanzania DHS data shows consistency with the national figure for women of this age group (33.8%) [8] in both the camp (36%) and the surrounding villages (32%), while for men aged 15–24 years condom use at last high-risk sex in the camps (44%) was similar to the national proportion (45.5%) and much lower in the surrounding villages (24%). Results in the camp may be due to good accessibility to condoms and high levels of awareness about HIV. However, there is clearly need to increase condom use overall. Although all youth engaging in high risk sex should be targeted, lower condom use at last sex reported in the villages points to the need for programs that ensure availability of condoms and strengthen appropriate behavior change strategies. As well, lower comprehensive correct knowledge of HIV/AIDS among younger refugees compared to village youth, and a very low proportion of refugee respondents with accepting attitudes towards people living with HIV/AIDS highlight key areas for improvement in HIV/AIDS education programs. Among those respondents who reported an STI symptom in the past 12 months, a greater proportion in villages, compared with the camp, sought treatment. There may be need for better integration of STI-related issues into ongoing health education programs and service delivery in the camp. Although the majority of respondents in both the camp and villages indicated they knew where to go for HIV testing, more refugees than village respondents had ever had an HIV test. This may indicate better accessibility of these services within the camp and the need for service improvements in the villages.

Several limitations should be considered in interpretation of study results. First, the survey captured the experience of fewer men than women in both the camp and surrounding villages, and male to female ratios differ between the camp (8.6 males for every 10 females) and village (7 males for every 10 females) populations. The greater proportion of males aged 15–24 years in the camp (48%) compared to the villages (30%) reflects a relative absence of older refugee males. This may be due to war-related deaths, participation in ongoing conflict, the need to stay behind to protect land, or migration to other areas for economic livelihood. It is not possible to interpret how this may have biased the results. Secondly, recall bias is an important limitation in any study that includes retrospective questions. Thirdly, re-sampling in the camp was necessary due to repatriation and the relocation of some households. There may have been a difference between the households that repatriated or relocated, and those included in the survey. However, according to UNHCR field staff, there were no specific characteristics that distinguished repatriated refugees from those who had not repatriated.

While the vast majority of refugees who reported transactional sex indicated that it occurred after displacement, this cannot be identified as occurring in the camp since respondents may have been displaced elsewhere prior to arrival at the Lugufu camp. Younger respondents (15–20 years) who reported transactional sex may have been very young (5–10 years) when they arrived to Lugufu, becoming sexually active as they matured while in the camp. As such, it is possible that transactional sex for some younger individuals could only have occurred after displacement, thereby creating a bias of results in the timing of transactional sex towards post-displacement. Similarly, this survey does not identify the age at forced sex for those respondents who reported that experience. Given that many refugees had been living in the camp for several years, it is difficult to ascertain the extent to which forced sex may be temporally associated with displacement.

Forced sex is a tragic hallmark of the ongoing conflict in the DRC. The World Health Organization (WHO) reports that SGBV is one of the greatest threats to women's health in that country and that by 2005 over 41,000 cases had been reported in four provinces since 1998 [18]. The WHO also estimates that in Tanzania one in ten women between the ages of 15–49 has experienced SGBV by a non-partner since the age of 15 years [19]. It is generally accepted that underreporting of this form of violence in quantitative research is far more likely than overreporting [20]. Furthermore, fear of retribution could have lead to under-reporting of forced sex by authority figures. Although interviewers were trained to address this issue with sensitivity, the forced sex results reported in Table 4 may be underreported for all respondents. It is possible that more focused training in this area would have helped interviewers elicit more complete information on forced sex.

## Conclusion

This research has clear programmatic implications. Several findings point to the need for interventions that address HIV-behaviors in the younger age groups, especially in the refugee population. This includes training, education, and life skills programs that emphasize the benefits of delayed sexual initiation, the risks involved in transactional sex, and the importance of correct and consistent condom use. These programs should also be made available to youth in the host villages, where condom use is fairly low.

Program planning should also consider the needs of girls and women in these populations. This includes further investigation into the impact of forced sex within regular partnerships and programs that encourage male involvement in addressing this issue. This study points to the need for programs to prevent intimate partner violence and care for survivors in both the camp and host villages. As well, study results highlight the vulnerability of widowed, divorced and never-married women. Program managers should verify that current commodity distribution systems ensure women access to resources, and consider additional program responses. Programs that focus on youth behavior change and participatory knowledge building have been developed in non-refugee settings with promising results, particularly in reducing male transactional sex [21]. Adaptations for refugee populations, especially in protracted situations, do exist but should be improved and prioritized.

Although both refugee and village respondents reported good accessibility of condoms, condom use patterns suggest behavioral barriers. Effective approaches to promote the use of condoms exist and should be a prominent part of programs in this setting. Marketplaces and small enterprises may be fruitful focal points for HIV prevention efforts aimed at both refugees and village populations since most interaction between populations occurs there. The smaller proportion of respondents in surrounding villages who had ever been tested for HIV, compared with refugee respondents, may highlight the inaccessibility of testing services in villages. Program managers should investigate barriers to HIV testing and expand services as needed.

Study results also offer direction for further research on factors that may increase or decrease HIV transmission in conflict-affected and displaced settings. Further investigation is needed to understand the dynamics between such environments and forced sex by a regular partner, as well as forced sex by others. This study did not include an assessment of health services available to refugee and village populations. This should be done to identify gaps in service delivery that can indirectly impact the spread of HIV. As well, research on war-related widowhood and spousal separation within the context of transactional sex, and research on the effectiveness of programmatic responses to this issue would mark an important contribution to understanding the impact of conflict and displacement on HIV transmission.

## Competing interests

The authors declare that they have no competing interests. ER led field work for this research under contract to UNHCR.

## Authors' contributions

PBS, MS, and NP conceived of the study and designed the protocol. ER led field work for this research and undertook analysis under contract to UNHCR, and wrote the paper. PBS provided critical interpretation of the intellectual content and drafting of the paper. ZT and GM participated in the management of field level data collection. All authors have read and approved the final manuscript.
